# Trade Agreements and Direct-to-Consumer Advertising of Pharmaceuticals

**DOI:** 10.15171/ijhpm.2017.124

**Published:** 2017-10-16

**Authors:** Deborah Gleeson, David B. Menkes

**Affiliations:** ^1^School of Psychology and Public Health, La Trobe University, Melbourne, Australia.; ^2^Waikato Clinical Campus, University of Auckland, Auckland, New Zealand.

**Keywords:** Trade Agreements, Pharmaceuticals, Advertising, Direct-to-Consumer Advertising (DTCA), Investor-State Dispute Settlement

## Abstract

There is growing international concern about the risks posed by direct-to-consumer advertising (DTCA) of prescription pharmaceuticals, including via the internet. Recent trade agreements negotiated by the United States, however, incorporate provisions that may constrain national regulation of DTCA. Some provisions explicitly mention DTCA; others enable foreign investors to seek compensation if new regulations are seen to harm their investments. These provisions may thus prevent countries from restricting DTCA or put them at risk of expensive legal action from companies seeking damages due to restrictions on advertising. While the most recent example, the Trans-Pacific Partnership Agreement (TPP), collapsed following US withdrawal in January 2017, early indications of the Trump Administration’s trade policy agenda signal an even more aggressive approach on the part of the United States in negotiating advantages for American businesses. Furthermore, the eleven remaining TPP countries may decide to proceed with the agreement in the absence of the United States, with most of the original text (including the provisions relevant to DTCA) intact.

## Background

International trade agreements negotiated over the last two decades include many provisions affecting domestic health policy,^1^ but their implications for direct-to-consumer advertising (DTCA) remain relatively unexplored. This paper examines their implications for the ability of nation states to prohibit or circumscribe DTCA.

## Why Direct-to-Consumer Advertising Is Contentious?

DTCA of prescription pharmaceuticals is banned in most countries due to perceived deleterious effects on rational prescribing, pharmaceutical expenditure, and health outcomes. DTCA increases expenditure by stimulating demand for particular, usually patented, products and shifting demand away from cheaper alternatives.^2^ DTCA is also associated with distorted drug information, unnecessary prescriptions, and reduced prescribing quality.^2,3^ Drugs promoted via DTCA are often early in their product lifecycle and sometimes subsequently manifest serious harms leading to market withdrawal.^4^ Various benefits have been claimed for DTCA, including patient empowerment, informed decision-making and reduced disease-related stigma.^2,5^ In support of this, there is some evidence that DTCA increases doctor visits by newly diagnosed patients and increases treatment of preventable conditions.^6^ The evidence base is not sufficiently developed, however, to establish whether these benefits outweigh the apparent harms of DTCA.^2,7^ Concerns that themotivation for DTCA is more about marketing than providing information to the public are reinforced by evidence that pharmaceutical companies spend nearly twice as much on marketing as on research and development.^8^

More recently, concerns have been expressed about how pharmaceutical companies are circumventing restrictions on DTCA by promoting ‘disease awareness’ while avoiding specific mention of their products.^9^ Similarly, direct to consumer marketing of medical testing has developed into a highly profitable variation on the theme of exploiting the public’s health anxieties and often unsophisticated understanding of risk.^10^ These developments indicate the industry’s creativity in identifying and exploiting weaknesses in DTCA regulation and suggest that continued vigilance is needed.

## Regulation of Direct-to-Consumer Advertising

Digital or internet-based DTCA - the type explicitly targeted in recently negotiated US trade agreements - raises particular challenges for regulators. This form of advertising is increasingly important to the pharmaceutical industry given the public’s extensive and growing use of the internet for health information.^11^ A study of violations and warnings sent by the US Food and Drug Administration (FDA) to pharmaceutical companies from 2005-2014 found the overwhelming majority (approximately 95%) of alleged breaches related to online advertising.^5^ Violations typically involved inadequate or misleading information regarding indications, efficacy and harms of advertised products.^5^

In the two industrialized countries which currently allow DTCA – the United States and New Zealand – momentum is building for policy change.^12,13^ Opposition comes from both professional and consumer groups, and is apparent also in countries resisting the proposed introduction of DTCA.^14^ Policy flexibility sacrificed during trade negotiations, however, may compromise efforts to restrict DTCA.

## Trade Agreements and Direct-to-Consumer Advertising

The first trade agreement to specifically mention DTCA was the Australia-US Free Trade Agreement (AUSFTA), which came into force in 2005. The United States, which hosts the headquarters for many of the world’s transnational pharmaceutical companies, sought to legalise DTCA via the internet through the clause^15^:

*“Each Party shall permit a pharmaceutical manufacturer to disseminate to health professionals and consumers through the manufacturer’s Internet site registered in the territory of the Party, and on other Internet sites registered in the territory of the Party linked to that site, truthful and not misleading information regarding its pharmaceuticals that are approved for sale in the Party’s territory as is permitted to be disseminated under the Party’s laws, regulations, and procedures, provided that the information includes a balance of risks and benefits and encompasses all indications for which the Party’s competent regulatory authorities have approved the marketing of the pharmaceuticals.*”

Despite the intent of this text to legalise DTCA, Australia was able to maintain its prohibition due to the phrase “as is permitted to be disseminated under the Party’s laws, regulations, and procedures,” which effectively neutralised the provision.^16^

The inclusion of the DTCA provision in the AUSFTA, however, had a further consequence. Trade agreements negotiated by the US tend to follow a ‘template approach,’ with the text of each agreement building on the last. When the United States negotiated its next trade agreement with South Korea, the US-Korea Free Trade Agreement (known as KORUS), the DTCA provision was included, but the crucial reference to existing domestic laws, regulations and procedures (“as is permitted to be disseminated under the Party’s laws, regulations and procedures”) was omitted.^16^

The net effect of KORUS on South Korea’s prohibition of pharmaceutical advertising is uncertain. In 2008, not long after the signing of KORUS the previous year, regulations were relaxed to allow advertising of prescription drug treatments for certain infectious diseases,^17^ but it is unclear to what extent these changes are related to the implementation of KORUS.

AUSFTA and KORUS set a new norm for the inclusion of DTCA clauses in US-led trade agreements – one which was carried through into the negotiations for the twelve-country Trans-Pacific Partnership Agreement (TPP).^16^ Negotiation of the TPP raised concerns about the health effects that could arise from, for example, expanded intellectual property protections for pharmaceuticals, rules applying to pharmaceutical coverage programs, and the inclusion of an investor-state dispute settlement mechanism allowing foreign investors to seek compensation over policies or laws that harm their investments.^18,19^ While the TPP apparently collapsed following the US withdrawal in January 2017, it remains important to analyse its implications for DTCA, given the US tendency to use previously negotiated text as the starting point for future trade agreements. The remaining 11 parties are also considering revitalising the agreement without the United States,^20^ and it is unclear to what degree the original text will be retained. The prime ministers of Japan and New Zealand, two countries which had already ratified the TPP prior to US withdrawal, appear intent on retaining the original text and avoiding re-negotiation.^21^

The DTCA clause in the TPP is similar to AUSFTA, at first glance appearing to legalise digital DTCA, but also making the clause subject to participating countries’ laws, regulations and procedures.^22^ The clause was not subject to the TPP’s state-to-state dispute settlement mechanisms, meaning that one country could not have enforced another’s implementation of the provision.

On the other hand, the TPP would have conferred an additional risk, in comparison with AUSFTA, for countries seeking to maintain or introduce restrictions on DTCA. An investor-state dispute settlement (ISDS) mechanism in TPP’s investment chapter enables a company incorporated in one nation to bring legal action against another’s government in an international tribunal, by arguing that a policy or law change harmed its investments.

In trade agreements with ISDS, the inclusion of DTCA provisions could be perceived as affecting investor rights. Government attempts to prohibit DTCA, or to regulate advertising aimed at prescribers, could thus be contested using ISDS. The attractiveness of ISDS to industry is illustrated by US pharmaceutical giant Eli Lilly’s (ultimately unsuccessful) case brought against Canada, seeking $500 million CAD in compensation after patents on two drugs were revoked by Canadian courts.^23^

Countries that prohibit DTCA have sought to neutralize the impact of pro-DTCA provisions through the clever use of language (as in the case of AUSFTA and the TPP), but the combination of these provisions with ISDS presents new risks. ISDS can be prohibitively expensive: the average cost of defending a case has been estimated at 8 million USD,^24^ and the awards can be in the hundreds of millions.^25^ In this context, even the threat of litigation can deter countries from adopting policies that impact on foreign investors.

It seems likely that having secured a DTCA clause in AUSFTA and KORUS, and having negotiated one for the TPP, the United States will continue to pursue this strategy in future negotiations. Early indications of the Trump Administration’s trade policy agenda^26^ herald an even more aggressive use of leverage on the part of the United States to secure access to foreign markets for US corporations. Furthermore, the provisions relevant to DTCA may well be adopted in their current form if the eleven remaining countries proceed with the TPP, particularly if as much as possible of the TPP text is retained in order to progress the agreement “expeditiously”^20^ and perhaps also in the hope that the United States will re-join the agreement in future.

## Conclusion

Provisions in recent trade agreements negotiated by the US threaten domestic regulation of pharmaceutical advertising. Countries wanting to impose or retain advertising restrictions should be cautious about accepting provisions that legalise DTCA, or that could lend weight to industry disputes over DTCA regulation – particularly in the presence of ISDS.

## Ethical issues

Not applicable.

## Competing interests

DG often represents the Public Health Association of Australia on matters regarding international trade agreements. She has accepted travel funds from various non-government (not-for-profit) and government sources to attend speaking events related to trade agreements. DBM serves on two NZ Government committees relevant to pharmacotherapy, and has been active in the International Society of Drug Bulletins (ISDB). The authors have no other competing interests to declare. The views expressed in this article are their own and not the views of any organisation with which they are affiliated.

## Authors’ contributions

Both authors contributed to the first draft of the paper, revised it for critical intellectual content and reviewed the final draft.

## Authors’ affiliations

^1^School of Psychology and Public Health, La Trobe University, Melbourne, Australia. ^2^Waikato Clinical Campus, University of Auckland, Auckland, New Zealand.
